# 4-Chloro­anilinium 2-carb­oxy-4,5-dichloro­benzoate

**DOI:** 10.1107/S160053680903044X

**Published:** 2009-08-08

**Authors:** Graham Smith, Urs D. Wermuth, Jonathan M. White

**Affiliations:** a School of Physical and Chemical Sciences, Queensland University of Technology, GPO Box 2434, Brisbane, Queensland 4001, Australia; b BIO-21 Molecular Science and Biotechnology, University of Melbourne, Parkville, Victoria 3052, Australia

## Abstract

The structure of the 1:1 proton-transfer compound of 4-chloro­aniline with 4,5-dichloro­phthalic acid (DCPA), *viz*.  C_6_H_7_ClN^+^·C_8_H_3_Cl_2_O_4_^−^, has been determined at 130 K. The non-planar hydrogen phthalate anions and the 4-chloro­anilinium cations form two-dimensional O—H⋯O and N—H⋯O hydrogen-bonded substructures which have no peripheral extension. Between the sheets there are weak π–π associations between alternating cation–anion aromatic ring systems [shortest centroid–centroid separation = 3.735 (4) Å].

## Related literature

For the structures of other hydrogen DCPA salts with aromatic Lewis bases showing similar two-dimensional substructures, see: Smith *et al.* (2008**b* ▶)*. This contrasts with the majority of the compounds in which the DCPA anion is planar with a short intra­molecular carboxylic acid hydrogen bond, see: Smith *et al.* (2007 ▶, 2008*a* ▶, 2009 ▶).
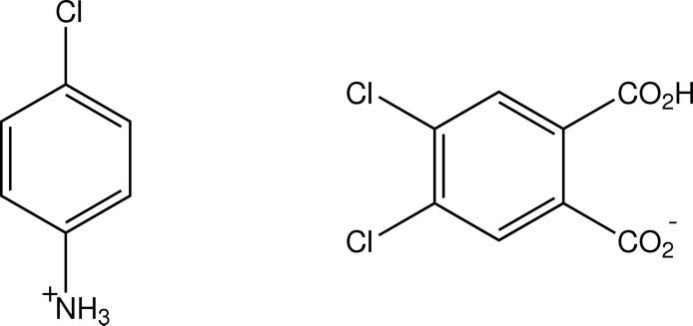


## Experimental

### 

#### Crystal data

C_6_H_7_ClN^+^·C_8_H_3_Cl_2_O_4_^−^
*M* *_r_* = 362.58
Monoclinic, 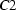

*a* = 12.8171 (8) Å
*b* = 7.5954 (3) Å
*c* = 16.0909 (6) Å
β = 109.815 (5)°
*V* = 1473.72 (13) Å^3^
*Z* = 4
Cu *K*α radiation
μ = 5.80 mm^−1^
*T* = 130 K
0.34 × 0.27 × 0.05 mm

#### Data collection

Oxford Diffraction Gemini Ultra CCD-detector diffractometer
Absorption correction: multi-scan (*SADABS*; Sheldrick, 1996 ▶) *T*_min_ = 0.201, *T*_max_ = 0.748
3680 measured reflections
2288 independent reflections
1879 reflections with *I* > 2σ(*I*)
*R*_int_ = 0.061

#### Refinement

*R*[*F* ^2^ > 2σ(*F* ^2^)] = 0.062
*wR*(*F* ^2^) = 0.175
*S* = 1.00
2288 reflections
215 parameters
1 restraint
H atoms treated by a mixture of independent and constrained refinement
Δρ_max_ = 0.64 e Å^−3^
Δρ_min_ = −0.56 e Å^−3^
Absolute structure: Flack (1983 ▶), 723 Friedel pairs
Flack parameter: 0.04 (3)

### 

Data collection: *CrysAlis CCD* (Oxford Diffraction, 2007 ▶); cell refinement: *CrysAlis RED* (Oxford Diffraction, 2007 ▶); data reduction: *CrysAlis RED*; program(s) used to solve structure: *SHELXS97* (Sheldrick, 2008 ▶); program(s) used to refine structure: *SHELXL97* (Sheldrick, 2008 ▶); molecular graphics: *PLATON* (Spek, 2009 ▶); software used to prepare material for publication: *PLATON*.

## Supplementary Material

Crystal structure: contains datablocks global, I. DOI: 10.1107/S160053680903044X/at2844sup1.cif

Structure factors: contains datablocks I. DOI: 10.1107/S160053680903044X/at2844Isup2.hkl

Additional supplementary materials:  crystallographic information; 3D view; checkCIF report

## Figures and Tables

**Table 1:** Hydrogen-bond geometry (Å, °)

*D*—H⋯*A*	*D*—H	H⋯*A*	*D*⋯*A*	*D*—H⋯*A*
O12—H12⋯O21^i^	0.81 (3)	1.69 (3)	2.485 (5)	166 (4)
N1*A*—H11*A*⋯O11^ii^	0.89 (4)	2.03 (4)	2.867 (8)	157 (4)
N1*A*—H11*A*⋯O22	0.89 (4)	2.59 (4)	2.875 (7)	100 (2)
N1*A*—H12*A*⋯O22	0.88 (3)	2.58 (5)	2.875 (7)	100 (3)
N1*A*—H12*A*⋯O22^iii^	0.88 (3)	1.94 (3)	2.813 (6)	172 (5)
N1*A*—H13*A*⋯O12^iv^	0.88 (4)	2.58 (5)	3.019 (7)	112 (3)
N1*A*—H13*A*⋯O21^iv^	0.88 (4)	1.94 (4)	2.805 (7)	166 (4)

Symmetry codes: (i) 
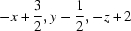
; (ii) 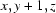; (iii) 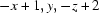; (iv) 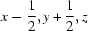.

**Fig. 1.**
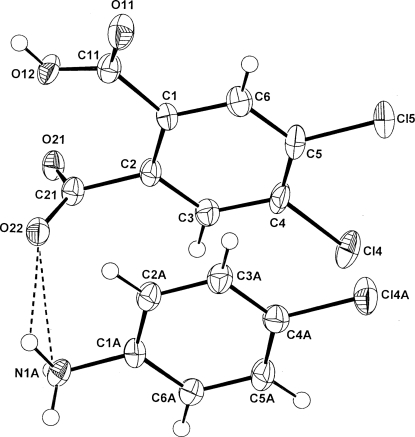
Molecular configuration and atom numbering scheme for the 4-chloroanilinium cation and the hydrogen 4,5-dichlorophthalate anion in (I). Non-H atoms are shown as 50% probability displacement ellipsoids. Hydrogen bonds are shown as dashed lines.

**Fig. 2.**
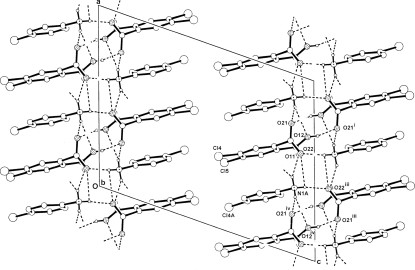
The two-dimensional sheet structure in (I) viewed down the b axis in the unit cell. Non-interactive H atoms are omitted and hydrogen bonds are shown as dashed lines. (For symmetry codes, see Table 1).

## supplementary crystallographic information

### Comment

Among the known structures of the 1:1 aromatic Lewis base compounds of 4,5-dichlorophthalic acid (DCPA), a small number have similar hydrogen-bonded substructures in which the basic *N*^+^—*H*···O_carboxyl_ linked cation–anion unit is propagated in two dimensions (Smith *et al.*, 2008*b*). In these the DCPA anions are non-planar with the carboxylic acid and carboxylate groups rotated out of the plane of the benzene ring. This contrasts with the majority of the compounds in which the DCPA anion is planar with a short intramolecular carboxylic acid *O*–*H*···O_carboxyl_ hydrogen bond (Smith *et al.*, 2007, 2008*a*, 2009). Since the examples having the two-dimensional substructures were substituted anilines, the 4-chloro-analogue was reacted with DCPA, giving anhydrous 4-chloroanilinium 2-carboxy-4,5-dichlorobenzoate C_6_H_7_ClN^+^. C_8_H_3_Cl_2_O_4_^-^ (I) and its structure is reported here.

In (I), as expected, the primary hydrogen-bonded cation–anion structural unit (Fig.1) provides the basis for formation of the previously described two-dimensional substructure which extends across the *ab* plane in the unit cell, having no lateral interactions (Fig. 2). The hydrogen bonding within the plane (Table 1) includes a cyclic *R*^2^_1_(6) anilinium–H···O12,O21 interaction. The alternating cation–anion aromatic ring systems (C1–C6 and C1A–C6A) give weak π–π interactions [shortest centroid separation 3.735 (4) Å]. The hydrogen 4,5-dichlorophthalate anion is non-planar [torsion angles C2–C1–C11–O11, 163.7 (7)°: C1–C2–C21–O22, -83.3 (8)°].,

### Experimental

The title compound (I) was synthesized by heating together 1 mmol quantities of 4-chloroaniline and 4,5-dichlorophthalic acid in 50 ml of 50% aqueous methanol for 10 min under reflux. After concentration to *ca*. 30 ml, partial room-temperature evaporation of the hot-filtered solution gave small colourless plates [m.p. 492 K]).

### Refinement

Hydrogen atoms potentially involved in hydrogen-bonding interactions were located by difference methods and their positional and isotropic displacement parameters were refined. Other H atoms were included in the refinement at calculated positions [C–H, 0.93 Å] and treated as riding models with *U*_iso_(H) = 1.2*U*_eq_ (C).

### Crystal data

**Table:**

C_6_H_7_ClN^+^·C_8_H_3_Cl_2_O_4_^−^	*F*(000) = 736
*M**_r_* = 362.58	*D*_x_ = 1.634 Mg m^−^^3^
Monoclinic, *C*2	Melting point: 492 K
Hall symbol: C 2y	Cu *K*α radiation, λ = 1.54184 Å
*a* = 12.8171 (8) Å	Cell parameters from 1721 reflections
*b* = 7.5954 (3) Å	θ = 2.9–71.8°
*c* = 16.0909 (6) Å	µ = 5.80 mm^−^^1^
β = 109.815 (5)°	*T* = 130 K
*V* = 1473.72 (13) Å^3^	Plate, colourless
*Z* = 4	0.34 × 0.27 × 0.05 mm

### Data collection

**Table:**

Oxford Diffraction Gemini Ultra CCD-detector diffractometer	2288 independent reflections
Radiation source: Enhance Ultra (Cu) X-ray source	1879 reflections with *I* > 2σ(*I*)
mirror	*R*_int_ = 0.061
ω scans	θ_max_ = 72.1°, θ_min_ = 2.9°
Absorption correction: multi-scan (*SADABS*; Sheldrick, 1996)	*h* = −15→14
*T*_min_ = 0.201, *T*_max_ = 0.748	*k* = −9→8
3680 measured reflections	*l* = −15→19

### Refinement

**Table:**

Refinement on *F*^2^	Secondary atom site location: difference Fourier map
Least-squares matrix: full	Hydrogen site location: inferred from neighbouring sites
*R*[*F*^2^ > 2σ(*F*^2^)] = 0.062	H atoms treated by a mixture of independent and constrained refinement
*wR*(*F*^2^) = 0.175	*w* = 1/[σ^2^(*F*_o_^2^) + (0.135*P*)^2^] where *P* = (*F*_o_^2^ + 2*F*_c_^2^)/3
*S* = 1.00	(Δ/σ)_max_ < 0.001
2288 reflections	Δρ_max_ = 0.64 e Å^−^^3^
215 parameters	Δρ_min_ = −0.56 e Å^−^^3^
1 restraint	Absolute structure: Flack (1983), 723 Friedel pairs
Primary atom site location: structure-invariant direct methods	Flack parameter: 0.04 (3)

### Special details

**Table:**

Geometry. Bond distances, angles *etc*. have been calculated using the rounded fractional coordinates. All su's are estimated from the variances of the (full) variance-covariance matrix. The cell e.s.d.'s are taken into account in the estimation of distances, angles and torsion angles
Refinement. Refinement of *F*^2^ against ALL reflections. The weighted *R*-factor *wR* and goodness of fit *S* are based on *F*^2^, conventional *R*-factors *R* are based on *F*, with *F* set to zero for negative *F*^2^. The threshold expression of *F*^2^ > σ(*F*^2^) is used only for calculating *R*-factors(gt) *etc*. and is not relevant to the choice of reflections for refinement. *R*-factors based on *F*^2^ are statistically about twice as large as those based on *F*, and *R*- factors based on ALL data will be even larger.

### Fractional atomic coordinates and isotropic or equivalent isotropic displacement parameters (Å^2^)

**Table:**

	*x*	*y*	*z*	*U*_iso_*/*U*_eq_	
Cl4	0.39571 (14)	0.8094 (3)	0.55613 (9)	0.0377 (5)	
Cl5	0.37318 (14)	0.3980 (3)	0.58289 (9)	0.0418 (5)	
O11	0.5642 (4)	0.3295 (7)	0.9286 (3)	0.0348 (14)	
O12	0.6750 (3)	0.5632 (6)	0.9688 (2)	0.0282 (11)	
O21	0.7198 (3)	0.9010 (6)	0.8943 (2)	0.0243 (10)	
O22	0.5704 (3)	0.9285 (6)	0.9364 (2)	0.0272 (13)	
C1	0.5441 (5)	0.5652 (8)	0.8257 (4)	0.0244 (16)	
C2	0.5583 (5)	0.7457 (8)	0.8141 (3)	0.0222 (16)	
C3	0.5118 (5)	0.8181 (9)	0.7299 (4)	0.0257 (16)	
C4	0.4522 (5)	0.7126 (9)	0.6581 (4)	0.0289 (18)	
C5	0.4402 (5)	0.5333 (9)	0.6707 (4)	0.0311 (18)	
C6	0.4861 (5)	0.4599 (9)	0.7537 (4)	0.0276 (17)	
C11	0.5956 (5)	0.4739 (9)	0.9139 (4)	0.0244 (17)	
C21	0.6212 (4)	0.8673 (7)	0.8897 (3)	0.0237 (16)	
Cl4A	0.13666 (14)	0.6564 (2)	0.60330 (9)	0.0387 (5)	
N1A	0.3688 (4)	1.1240 (7)	0.9059 (3)	0.0234 (14)	
C1A	0.3149 (5)	1.0096 (8)	0.8304 (3)	0.0233 (16)	
C2A	0.2717 (5)	1.0813 (9)	0.7460 (4)	0.0292 (17)	
C3A	0.2162 (5)	0.9729 (9)	0.6753 (4)	0.0300 (18)	
C4A	0.2064 (5)	0.7965 (9)	0.6903 (4)	0.0286 (17)	
C5A	0.2510 (5)	0.7210 (9)	0.7743 (4)	0.0283 (17)	
C6A	0.3054 (5)	0.8316 (9)	0.8443 (3)	0.0263 (18)	
H3	0.52050	0.93760	0.72140	0.0310*	
H6	0.47830	0.34000	0.76170	0.0340*	
H12	0.700 (3)	0.509 (4)	1.015 (2)	0.042 (11)*	
H2A	0.27990	1.20080	0.73710	0.0340*	
H3A	0.18610	1.01900	0.61860	0.0360*	
H5A	0.24450	0.60100	0.78290	0.0340*	
H6A	0.33560	0.78560	0.90100	0.0320*	
H11A	0.425 (3)	1.179 (4)	0.897 (3)	0.035 (10)*	
H12A	0.393 (4)	1.059 (3)	0.954 (2)	0.041 (11)*	
H13A	0.320 (4)	1.202 (4)	0.910 (3)	0.038 (11)*	

### Atomic displacement parameters (Å^2^)

**Table:**

	*U*^11^	*U*^22^	*U*^33^	*U*^12^	*U*^13^	*U*^23^
Cl4	0.0444 (9)	0.0546 (10)	0.0141 (6)	−0.0005 (8)	0.0035 (5)	0.0056 (6)
Cl5	0.0482 (10)	0.0479 (10)	0.0206 (6)	−0.0014 (8)	0.0002 (6)	−0.0120 (6)
O11	0.038 (2)	0.035 (3)	0.026 (2)	−0.011 (2)	0.0037 (17)	0.0002 (19)
O12	0.031 (2)	0.034 (2)	0.0147 (17)	0.0000 (18)	0.0015 (15)	0.0039 (16)
O21	0.0266 (18)	0.031 (2)	0.0155 (15)	−0.0022 (18)	0.0075 (14)	−0.0015 (15)
O22	0.026 (2)	0.033 (3)	0.0203 (17)	0.0019 (17)	0.0050 (15)	−0.0038 (16)
C1	0.028 (3)	0.027 (3)	0.017 (2)	0.001 (2)	0.006 (2)	−0.004 (2)
C2	0.023 (3)	0.029 (3)	0.015 (2)	−0.001 (2)	0.007 (2)	−0.001 (2)
C3	0.028 (3)	0.029 (3)	0.020 (2)	0.001 (3)	0.008 (2)	−0.001 (2)
C4	0.029 (3)	0.043 (4)	0.011 (2)	0.002 (3)	0.002 (2)	0.000 (2)
C5	0.030 (3)	0.041 (4)	0.017 (2)	−0.005 (3)	0.001 (2)	−0.006 (3)
C6	0.030 (3)	0.031 (3)	0.024 (3)	−0.003 (3)	0.012 (2)	−0.002 (2)
C11	0.022 (3)	0.033 (3)	0.019 (3)	−0.003 (2)	0.008 (2)	−0.001 (2)
C21	0.023 (3)	0.030 (3)	0.018 (2)	−0.002 (2)	0.007 (2)	−0.001 (2)
Cl4A	0.0489 (9)	0.0436 (10)	0.0201 (6)	−0.0072 (8)	0.0073 (6)	−0.0054 (6)
N1A	0.023 (2)	0.030 (3)	0.016 (2)	0.001 (2)	0.0050 (16)	0.0040 (19)
C1A	0.024 (3)	0.029 (3)	0.017 (2)	−0.004 (2)	0.007 (2)	−0.003 (2)
C2A	0.036 (3)	0.029 (3)	0.020 (3)	0.005 (3)	0.006 (2)	0.001 (2)
C3A	0.034 (3)	0.039 (4)	0.016 (2)	0.000 (3)	0.007 (2)	0.001 (2)
C4A	0.035 (3)	0.029 (3)	0.022 (3)	0.003 (3)	0.010 (2)	0.000 (2)
C5A	0.032 (3)	0.031 (3)	0.021 (3)	−0.002 (3)	0.008 (2)	0.002 (2)
C6A	0.029 (3)	0.032 (4)	0.018 (2)	0.000 (3)	0.008 (2)	0.002 (2)

### Geometric parameters (Å, °)

**Table:**

Cl4—C4	1.718 (6)	C2—C3	1.395 (8)
Cl5—C5	1.723 (7)	C3—C4	1.401 (9)
Cl4A—C4A	1.744 (7)	C4—C5	1.393 (10)
O11—C11	1.219 (9)	C5—C6	1.381 (9)
O12—C11	1.291 (8)	C3—H3	0.9300
O21—C21	1.267 (7)	C6—H6	0.9300
O22—C21	1.240 (6)	C1A—C6A	1.383 (9)
O12—H12	0.81 (3)	C1A—C2A	1.392 (8)
N1A—C1A	1.463 (7)	C2A—C3A	1.389 (9)
N1A—H13A	0.89 (4)	C3A—C4A	1.375 (10)
N1A—H12A	0.88 (3)	C4A—C5A	1.400 (9)
N1A—H11A	0.88 (4)	C5A—C6A	1.389 (8)
C1—C2	1.404 (9)	C2A—H2A	0.9300
C1—C11	1.515 (9)	C3A—H3A	0.9300
C1—C6	1.397 (9)	C5A—H5A	0.9300
C2—C21	1.523 (7)	C6A—H6A	0.9300
			
C11—O12—H12	110 (2)	O22—C21—C2	117.9 (5)
H11A—N1A—H12A	110 (4)	O21—C21—C2	114.6 (5)
H11A—N1A—H13A	109 (4)	C2—C3—H3	120.00
C1A—N1A—H13A	108 (3)	C4—C3—H3	120.00
C1A—N1A—H11A	109 (3)	C5—C6—H6	120.00
H12A—N1A—H13A	111 (4)	C1—C6—H6	120.00
C6—C1—C11	117.1 (6)	C2A—C1A—C6A	120.9 (5)
C2—C1—C11	122.5 (5)	N1A—C1A—C6A	119.3 (4)
C2—C1—C6	120.4 (5)	N1A—C1A—C2A	119.8 (5)
C1—C2—C3	118.8 (5)	C1A—C2A—C3A	119.4 (6)
C3—C2—C21	118.2 (5)	C2A—C3A—C4A	119.1 (6)
C1—C2—C21	122.9 (5)	Cl4A—C4A—C3A	120.4 (5)
C2—C3—C4	120.6 (6)	Cl4A—C4A—C5A	117.1 (5)
C3—C4—C5	119.7 (6)	C3A—C4A—C5A	122.5 (6)
Cl4—C4—C5	121.7 (5)	C4A—C5A—C6A	117.6 (6)
Cl4—C4—C3	118.6 (5)	C1A—C6A—C5A	120.5 (5)
Cl5—C5—C6	118.9 (5)	C1A—C2A—H2A	120.00
C4—C5—C6	120.2 (6)	C3A—C2A—H2A	120.00
Cl5—C5—C4	120.9 (5)	C2A—C3A—H3A	120.00
C1—C6—C5	120.2 (6)	C4A—C3A—H3A	120.00
O11—C11—C1	121.7 (6)	C4A—C5A—H5A	121.00
O11—C11—O12	125.1 (6)	C6A—C5A—H5A	121.00
O12—C11—C1	113.1 (6)	C1A—C6A—H6A	120.00
O21—C21—O22	127.4 (5)	C5A—C6A—H6A	120.00
			
C6—C1—C2—C3	1.2 (10)	C2—C3—C4—C5	−0.7 (10)
C6—C1—C2—C21	−179.6 (6)	Cl4—C4—C5—Cl5	3.1 (9)
C11—C1—C2—C3	177.4 (6)	Cl4—C4—C5—C6	−179.6 (5)
C11—C1—C2—C21	−3.4 (10)	C3—C4—C5—Cl5	−176.9 (5)
C2—C1—C6—C5	−1.4 (10)	C3—C4—C5—C6	0.5 (10)
C11—C1—C6—C5	−177.8 (6)	Cl5—C5—C6—C1	177.9 (5)
C2—C1—C11—O11	163.7 (7)	C4—C5—C6—C1	0.5 (10)
C2—C1—C11—O12	−17.4 (9)	N1A—C1A—C2A—C3A	−177.1 (6)
C6—C1—C11—O11	−20.0 (10)	C6A—C1A—C2A—C3A	1.5 (10)
C6—C1—C11—O12	159.0 (6)	N1A—C1A—C6A—C5A	177.6 (6)
C1—C2—C3—C4	−0.2 (10)	C2A—C1A—C6A—C5A	−0.9 (10)
C21—C2—C3—C4	−179.4 (6)	C1A—C2A—C3A—C4A	−0.8 (10)
C1—C2—C21—O21	100.2 (7)	C2A—C3A—C4A—Cl4A	179.7 (5)
C1—C2—C21—O22	−83.3 (8)	C2A—C3A—C4A—C5A	−0.5 (10)
C3—C2—C21—O21	−80.6 (7)	Cl4A—C4A—C5A—C6A	−179.1 (5)
C3—C2—C21—O22	95.9 (7)	C3A—C4A—C5A—C6A	1.0 (10)
C2—C3—C4—Cl4	179.4 (5)	C4A—C5A—C6A—C1A	−0.3 (10)

### Hydrogen-bond geometry (Å, °)

**Table:**

*D*—H···*A*	*D*—H	H···*A*	*D*···*A*	*D*—H···*A*
O12—H12···O21^i^	0.81 (3)	1.69 (3)	2.485 (5)	166 (4)
N1A—H11A···O11^ii^	0.89 (4)	2.03 (4)	2.867 (8)	157 (4)
N1A—H11A···O22	0.89 (4)	2.59 (4)	2.875 (7)	100 (2)
N1A—H12A···O22	0.88 (3)	2.58 (5)	2.875 (7)	100 (3)
N1A—H12A···O22^iii^	0.88 (3)	1.94 (3)	2.813 (6)	172 (5)
N1A—H13A···O12^iv^	0.88 (4)	2.58 (5)	3.019 (7)	112 (3)
N1A—H13A···O21^iv^	0.88 (4)	1.94 (4)	2.805 (7)	166 (4)
C5A—H5A···O21^v^	0.93	2.45	3.212 (8)	139

Symmetry codes: (i) −*x*+3/2, *y*−1/2, −*z*+2; (ii) *x*, *y*+1, *z*; (iii) −*x*+1, *y*, −*z*+2; (iv) *x*−1/2, *y*+1/2, *z*; (v) *x*−1/2, *y*−1/2, *z*.
